# Growth performance and nutrient digestibility of beef heifers fed sorghum silage processed at harvest and stored for less than five months or over one year

**DOI:** 10.1093/tas/txag028

**Published:** 2026-03-28

**Authors:** Leandro O Abdelhadi, Guillermo A Mattioli, Fredric N Owens, Nicolás DiLorenzo

**Affiliations:** Facultad de Ciencias Veterinarias, Universidad Nacional de La Plata (UNLP), Buenos Aires, Argentina; North Florida Research and Education Center, Institute of Food and Agricultural Sciences, University of Florida, Marianna, FL, 32446-7906, United States; Facultad de Ciencias Veterinarias, Universidad Nacional de La Plata (UNLP), Buenos Aires, Argentina; Regents Professor Emeritus, Oklahoma State University, River Falls, WI, 54022, United States; North Florida Research and Education Center, Institute of Food and Agricultural Sciences, University of Florida, Marianna, FL, 32446-7906, United States

**Keywords:** backgrounding performance, degradability, digestibility, processing, sorghum silage, storage time

## Abstract

This study evaluated the effects of feeding sorghum silage (**SS**), either processed (**PRO**) or not (**NPR**) at harvest, on animal performance, apparent total tract digestibility (**ATTD**), and in situ nutrient degradability (**ISND**). The sorghum plant was harvested when the kernels were at the hard dough stage and kernel processing was applied or not prior to ensiling. Kernel processing was done with 110-tooth rolls, at 1.1 mm gap, and 50% speed differential, yielding a theoretical length of cut of 15 mm. On a dry matter (DM) basis, diets contained 90.5% SS, 6.5% soybean expeller, and 3% vitamin-mineral supplement. Performance and ATTD trials were conducted over two consecutive years using SS stored for < 150 days (**Y1**) or > 375 days (**Y2**). In situ nutrient digestibility was assessed on samples ensiled from 0 to 455 d. Animal performance studies were conducted using 96 Angus heifers each year in a completely randomized design with 6 pens (8 heifers/pen in both years), followed by an experiment to determine ATTD using one randomly selected heifer/pen at the end of each performance study. Data were analyzed as a completely randomized design using the Mixed procedure of SAS, with treatment as a fixed effect and initial body weight as covariate in the performance trials, and repeated measures in the ISND trial. In Y1, no differences in dry matter intake (DMI) were detected between treatments, but PRO improved final body weight and average daily gain (ADG; *P* < .02), as well as gain-to-feed ratio (*P* = .03). Apparent total tract digestibility of starch in Y1 was greater in PRO (*P* < .01), while no treatment effects were observed for NDF, ADF, or CP digestibility. In Y2, PRO increased DMI (*P* = .01), but ADG and feed efficiency were not different between treatments (*P* > .10). Nutrient intake and digestibility in Y2 were unaffected by treatments. For ISND, a treatment × day interaction was detected (*P* < .01) for DM degradability, where PRO increased DM degradability at d 66 and 202 of storage (*P* < .05). Starch degradability improved with storage time independently of processing. In conclusion, kernel processing of SS improved ADG by 15%, feed efficiency by 21%, and ATTD of starch by 8%, when compared to NPR in silage sored for < 150 d. However, processing did not provide any benefits when SS was stored > 375 days. Processing appears to enhance in situ DM degradability up to ∼300 days of storage, while starch degradability increased with storage time regardless of processing.

## Teaser

Processing a grain sorghum crop harvested as whole-plant silage to be fed within 150 days of harvest increased starch digestibility and beef cattle performance. However, the effect of processing at harvest on animal performance is greatly decreased if the same silage crop is stored for a longer time (≥375 d) prior to being fed.

## Introduction

Climate change can be expected to alter availability of feed ingredients for livestock production systems. Growing sorghum can ensure silage production regardless of water limitation or excess, elevated temperatures and limited soil fertility, specific conditions under which successful corn crop likely will be limited or uncertain (Gomez et al. 2025). One of the most important factors that limits sorghum silage (SS) quality and value is its low starch digestibility resulting from a combination of small berries and a dense proteinaceous matrix of prolamines in the peripheral endosperm layer of the kernel (Duodu et al. 2003). These factors reduce starch exposure, thereby limiting animal performance potentially derived from the grain portion of sorghum silage. When SS was processed prior to feeding to break the grains, starch digestion was improved and performance of beef heifers and steers was increased when compared with non-processed silage basal diets (Smith 1986). With new harvesting technologies, we now can process the full plant when harvested (chopped) for ensiling; hence, the starch in the grains should be more available for digestion. Besides, when SS was processed at harvest, the performance of backgrounded steers was similar to that achieved by steers fed corn silage; at 40% of the diet dry matter (Abdelhadi and Santini 2006). Additionally, Podversich et al. (2025) showed a positive effect of kernel processing on total tract digestibility of starch in heifers fed sorghum silage. Although the importance of grain processing of sorghum is widely known for maximum animal production (McCuistion et al. 2019) at the farm level, yet most SS is not kerned processed before feeding. The beneficial effect of longer storage time on increasing starch digestibility was demonstrated during in vitro and in situ trials using corn silage and high moisture corn (Daniel et al. 2015), and more recently in situ using whole-plant sorghum silage (Fernandes et al. 2020). Likewise, longer storage time typically increases starch digestibility of ensiled corn and sorghum grains.

Thus, the current research had three primary objectives; (i) to determine the impact of processing SS at harvest on cattle performance and apparent total tract digestibility (ATTD) of nutrients using growing-finishing beef cattle fed diets based on SS with a shorter storage time (<150 d); (ii) to determine the effects of feeding SS based diets with a longer storage time (>375 d) on animal performance and ATTD by beef cattle; and (iii) to evaluate changes over storage on nutrient composition and in situ (ruminal) digestion of SS with or without processing.

## Materials and methods

All procedures involving animals were approved by the Facultad de Ciencias Veterinarias—Universidad Nacional de La Plata, Buenos Aires, Argentina (Protocol #127-2-22 T—CICUAL), and by the University of Florida Institutional Animal Care and Use Committee (Protocol #202200000442—IACUC).

The research was conducted during three consecutive years and involved five experiments. The first-year performance study was run from June to September 2021 followed by a trial to determine ATTD of nutrients. The second-year performance trial lasted from June to August 2022 and was followed by the second ATTD trial; both at El Encuentro experimental farm, Buenos Aires, Argentina (35°23′ S; 58°25′ W). The final trial started with sorghum silage being sampled directly from the bag until 455 d of storage (from May 17, 2021 to August 15, 2022 at El Encuentro experimental farm, Buenos Aires, Argentina) and ended with the ISND trial conducted from January to February of 2023 at the University of Florida, North Florida Research and Education Center, in Marianna, FL, USA.

### Harvested treatments and storage

All experiments started with the seeding of dual-purpose sorghum for silage (ADV2450IG hybrid, Advanta Seeds, Irving-TX) on November 30, 2020 at El Encuentro Experimental farm; Buenos Aires, Argentina. Eleven hectares were seeded in north-south rows spaced at 35 cm in soil consisting of 90% fine, mixed, thermic Typic Argiudoll and 10% fine, illitic, thermic Petrocalcic Paleudoll (pH = 6.5, organic matter = 2.8%, NO_3_-*N* = 3.1 ppm, *P* = 7.2 ppm) seeded at 200.000 seeds/ha; with 50 kg/ha of diammonium phosphate included at seeding. After seeding, weed control used pre-emergence herbicides [150 mL/ha of imidazolinone (OnDuty, BASF, Buenos Aires, Argentina) plus 2 L/ha of atrazine]. When sorghum reached the six-leaf stage, 100 kg/ha of urea was applied. The amount of rainfall from seeding to harvest was 380 mm, with an additional 30 mm of total rainfall from soil preparation (October) until seeding.

Harvest occurred on May 17, 2021, at the hard-dough stage of maturity, with a precision chop harvester (Claas Jaguar 930, Harsewinkel, Germany). At harvest, two treatments were applied: crop processed at harvest (**PRO)** using a specific SS processor (rolls of 196 mm diameter, 660 mm wide, with 110 teeth spaced 2 mm each, 50% speed differential, and 1.1 mm roll gap; Metalurgica Perotti, San Francisco, Cordoba, Argentina); or not processed at harvest (**NPR)** achieved by the removal of the processor from the chopper.

Sorghum whole-plant was chopped at 15 mm theoretical length of cut, ensiled in two white polyethylene ag-bags (one for each processing treatment) of 60 m long × 2.74 m diameter with 220 µm of plastic thickness. Bags were stored for a total of 455 d; during which the PRO and NPR were fed in individual experiments of performance and ATTD in year 1 (Y1) and year 2 (Y2).

### Performance trials

On Y1, the feeding trial was run using PRO or NPR SS stored from 47 to 127 d, at El Encuentro Experimental farm facilities, using Angus weaned heifers. Including adaptation to diets and management for 17 d, the feeding trial lasted from July 3 to September 21, 2021 (80 d). On Y2, the feeding trial was run using the same SS but stored for a longer time (376 to 448 d) using the same facilities and animal type but using a different set of heifers from the same operation, in a trial that lasted 72 days (May 28 to August 8, 2022), including 12 days of adaptation to diets and management. Each year, heifers were grazing forage sorghum prior to entering the feedlot pens for the study thus, the adaptation was mainly to facilities and management. Heifers were fed their experimental diets during the adaptation once they entered the feedlot.

#### Experimental design and statistical analyses

In both feeding trials, 96 Angus weaned heifers (7 to 9 mo of age) were used each year in a completely randomized design (CRD) with 6 pens of 8 heifers per treatment. A mixed model of analysis in SAS was used that included the fixed effect of treatment (SS PRO or NPR) with initial BW as a covariate. For all the analyses, pen was considered the experimental unit. Significance was declared at *(P* ≤ .05). To avoid confounding effects of year and storage time, each year’s performance and digestibility trials were analyzed as separate experiments.

#### Diets and management

Each year, at weaning, heifers were managed following the health program of the farm which included: deworming with 1 mL/22.5 kg BW of levamisole (product concentration: 20 g/100 mL); a micro mineral dose of 3 mL/heifer (product concentration: 1.5 g Cu, 5 g Zn and 0.5 g Se in 100 mL); and external parasite control with 10 mL pour-on (product concentration: 5 g cypermethrin, 2 g carbaryl and 7 g piperonyl butoxide per 100 mL).

After applying health program, heifers were weighed for Year 1 (shrunk BW = 168.9 ± 20.7 kg) and Year 2 (shrunk BW = 193.0 ± 24.3 kg), respectively; stratified by weight, and assigned to a pen. Experimental pens were constructed with metal panels (Farmquip, Acebal, Sta. Fe, Argentina), in pens of 105 m^2^ each (8.43 × 12.45 m) allowing 13 m^2^/heifer. Each pen had 8 m of linear concrete bunk space (100 cm of linear bunk space per animal), a 3-m concrete apron after the bunk, and the rest of the pen area was covered using silty clayey gravel and cement (10 kg/m^2^).

For both years, diets contained [on a dry matter (DM) basis], 90.5% SS PRO or NPR, 6.5% soybean expeller and 3% mineral supplement (ROC90, Provimi, Argentina) as ingredients (Tables 1 and 5); were used. Experimental diets were delivered once daily at 0800 h using a tractor-pulled mixer equipped with ± 5 kg precision scale (Mixer Senor 228-10, Roldan, Sta. Fe, Argentina). Samples of ration ingredients as well as mixed ration were collected weekly and were frozen at −20°C until further laboratory analyses.

**Table 1:** Chemical composition of ingredients used in diets fed during performance and apparent digestion trials in the first year.

Item^a^	Ingredients^b^			
	SSNPR	SSPRO	SBE	Vitamin and mineral premix
**DM, % as fed**	32.5	33.7	96.0	95.3
**OM, % of DM**	92.7	92.5	93.9	52.1
**CP, % of DM**	8.6	8.6	44.1	93.9
**aNDF, % of DM**	37.2	39.1	10.0	9.2
**ADF, % of DM**	25.8	28.6	7.4	2.7
**Starch, % of DM**	28.9	27.0	0.9	3.3
**Lignin, % of DM**	5.8	6.8	0.4	1.2
**Fat, % of DM**	2.3	2.3	7.7	2.8
**Ash, % of DM**	7.3	7.5	6.1	47.9
**Ca, % of DM**	0.2	0.2	0.3	14.6
**P, % of DM**	0.2	0.2	0.6	0.2
**TDN, % of DM**	65.5	63.5	88.1	74.4
***Particle size, % of as fed retained* ^c^**				
**19.0 mm**	2.3	2.0	–	–
**8.0 mm**	59.0	51.9	–	–
**1.18 mm**	36.9	43.2	–	–
**Botton pan**	1.9	2.9	–	–

a Rock River Laboratory, Watertown-WI: DM, dry matter; OM, organic matter; CP, crude protein; EE, ether extract; aNDF, neutral detergent fiber using a-amylase; ADF, acid detergent fiber; Ca, calcium; P, phosphorus; TDN, total digestible nutrients.

b Ingredients: sorghum silage stored from 47 to 134 d, non-processed (SSNPR) or processed (SSPRO) at ensiling time; SBE, soybean expeller; Premix, mineral-vitamin (ROC90, Provimi, Argentina).

c Particle size was measured using the Penn State Particle Size Separator (Nasco, Fort Atkinson, WI) as described by Kononoff et al. (2003).

Throughout the feeding trial, diets were adjusted daily to a target bunk score of 0.5, using the SDSU 4-point bunk scoring system, where: 0 = empty bunk; 0.5 = thin layer of feed; 1 = thick layer of feed; 2 = 25–50% of feed offered; 3 = > 50% of feed offered. With a target bunk score of 0.5, adjustments on daily feed delivery were made as follows: for score 0, increased 4 kg/pen as fed, and for score 1, reduced 4 kg/pen. Feed was delivered, orts were removed, weighed, and discarded, and bunk scores were manually recorded. Weekly feed samples were collected to determine DM intake and were frozen at −20°C for subsequent analyses. Final diets fed to heifers are reported in Tables 2 and 6, for Y1 and Y2, respectively. Animals had free access to water during adaptation and experimental periods, while the mineral supplement had 90% CP, 15% Ca, 2.4% Na, 4% Cl, 1100 ppm Mn, 1250 ppm Zn, 340 ppm Cu, 5 ppm Se, 3.4 ppm Co, 19 ppm I, 1000 ppm of monensin, 75,000 IU/kg Vit. A, 7500 IU/kg Vit. D and 250 IU/kg Vit. E (ROC 90, Provimi, Argentina).

**Table 2:** Final composition of diets fed during performance and apparent digestion trials in the first year.

**Item** ^a^	**TMR Performance** ^b^		TMR Digestion	
	NPR	PRO	NPR	PRO
**DM, % as fed**	35.9	37.8	37.4	37.9
**OM, % of DM**	90.8	91.5	89.9	91.6
**CP, % of DM**	14.3	12.3	14.0	14.1
**aNDF, % of DM**	37.6	40.7	40.3	39.2
**ADF, % of DM**	25.7	26.9	28.7	27.3
**Starch, % of DM**	26.0	26.3	28.2	26.5
**isSD 0 h, %**	4.0	34.1	–	–
**isSD 7 h, %**	35.6	44.7	–	–
**Lignin, % of DM**	5.3	6.2	5.9	5.8
**Fat, % of DM**	2.9	2.7	2.4	2.5
**Ash, % of DM**	9.2	8.6	10.1	8.4
**Ca, % of DM**	0.7	0.6	0.5	0.5
**P, % of DM**	0.2	0.2	0.2	0.2
**TDN, % of DM**	60.5	63.7	65.4	63.9
**NEm, Mcal/kg^c^**	1.23	1.35	1.40	1.36
**NEg, Mcal/kg**	0.54	0.59	0.62	0.59

a Rock River Laboratory, Watertown-WI: DM, dry matter; OM, organic matter; CP, crude protein; EE, ether extract; aNDF, neutral detergent fiber using a-amylase; ADF, acid detergent fiber; isSD 0 h, ruminal in situ starch disappearance at 0 h, isSD 7 h, ruminal in situ starch disappearance at 7 h; Ca, calcium; P, phosphorus; TDN, total digestible nutrients.

b TMR, total mixed rations: NPR (non-processed) or PRO (processed) sorghum silage stored from 47 to 134 d included at 90.5% diet; soybean expeller included at 6.5% diet; vitamin and mineral premix (ROC90, Provimi, Argentina), included at 3% diet; all on DM basis.

c NEm, net energy for maintenance; NEg, net energy for gain: estimated using equations from Galyean et al. (2016).

**Table 3:** Effects of processing sorghum silage at harvest, on performance of beef heifers during the first-year trial.

**Item** ^a^	**Treatments** ^b^		**SEM** ^c^	***P*-value** ^d^
	NPR	PRO		
**Pens, n**	6	6	**-**	**-**
**DMI, kg/d**	8.42	8.09	0.284	0.44
**DMI, % of BW**	3.75	3.50	0.142	0.24
**Initial BW, kg**	182.7	182.5	9.511	0.99
**Final BW, kg**	239.4	246.8	1.822	0.02
**ADG, kg**	0.892	1.028	0.025	<0.01
**GTF, kg: kg**	0.107	0.128	0.006	0.03

a DMI, dry matter intake; BW, body weight; ADG, average daily gain; GTF, gain to feed ratio on a DM basis.

b Treatments: sorghum silage stored from 47 to 134 d non-processed (NPR) or processed (PRO) included at 90.5% of total diet; soybean expeller at 6.5% of total diet; vitamin and mineral premix (ROC90, Provimi, Argentina) at 3% of total diet; all on DM basis.

c Standard error of the mean (*n* = 6 pens/treatment).

d Observed significance level for treatment (*n* = 6 pens/treatment).

**Table 4:** Effects of processing sorghum silage on intake and apparent total tract digestibility of nutrients in backgrounding beef heifers during the first-year trial.

**Item** ^a^	**Treatments** ^b^		**SEM** ^c^	***P*-value** ^d^
	NPR	PRO		
**Heifers, n**	6	6	*-*	*-*
**Body weight, kg**	239.4	246.8	1.822	0.02
**Intake, kg/d**				
**DM**	8.97	7.55	0.544	0.10
**OM**	8.22	6.79	0.490	0.07
**NDF**	3.51	3.05	0.222	0.17
**ADF**	2.45	2.16	0.141	0.17
**CP**	1.27	1.06	0.092	0.15
**Starch**	2.53	1.99	0.151	0.03
**Change in NDF, %^e^**	5.99	0.97	1.944	0.10
**Fecal starch, %**	8.69	4.46	0.675	<0.01
**Digestibility, %**				
**DM**	54.74	60.45	5.163	0.45
**OM**	58.23	64.50	4.686	0.37
**NDF**	37.70	44.06	7.855	0.58
**ADF**	20.39	29.90	7.576	0.40
**CP**	50.43	59.02	4.656	0.22
**Starch**	86.48	93.55	1.103	<0.01

a DMI, dry matter; OM, organic matter; NDF, neutral detergent fiber; ADF, acid detergent fiber; CP, crude protein.

b Treatments: sorghum silage stored from 47 to 134 d non-processed (NPR) or processed (PRO) included at 90.5% of total diet; soybean expeller at 6.5% of total diet; vitamin and mineral premix (ROC90, Provimi, Argentina) at 3% of total diet; all on DM basis.

c Standard error of the mean (*n* = 6 pens/treatment).

d Observed significance level for treatment (*n* = 6 pens/treatment).

e Change in NDF, % = (NDF concentration refusals—NDF concentration diet)/NDF concentration diet × 100.

**Table 5:** Chemical composition of ingredients used in diets fed during performance and apparent digestion trials across the second year.

**Item** ^a^	**Ingredients** ^b^			
	SSNPR	SSPRO	SBE	Vitamin and mineral premix
**DM, %**	31.3	32.5	90.8	97.7
**OM, %**	91.3	91.4	93.8	51.9
**CP, %**	8.4	8.5	44.8	94.2
**EE, %**	2.1	2.0	6.9	2.6
**aNDF, %**	39.8	39.6	9.7	9.0
**ADF, %**	27.9	28.3	6.9	2.5
**Starch, %**	27.1	27.9	1.1	3.1
**Lignin, %**	6.2	6.9	0.3	1.1
**Ash, %**	7.8	8.0	6.0	48.3
**Ca, %**	0.17	0.18	0.27	14.4
**P, %**	0.18	0.19	0.51	0.24
**TDN, %**	63.9	62.8	86.8	73.9
***Particle size, % of as fed retained* ^c^**				
**19.0 mm**	1.1	1.1	–	–
**8.0 mm**	53.4	48.2	–	–
**1.18 mm**	42.6	46.9	–	–
**Botton pan**	2.9	3.7	–	–

a Rock River Laboratory, Watertown-WI: DM, dry matter; OM, organic matter; CP, crude protein; EE, ether extract; aNDF, neutral detergent fiber using a-amylase; ADF, acid detergent fiber; Ca, calcium; P, phosphorus; TDN, total digestible nutrients.

b Ingredients: sorghum silage stored from 376 to 455 d, non-processed (SSNPR) or processed (SSPRO) at ensiling time; SBE, soybean expeller; vitamin and mineral premix (ROC90, Provimi-Argentina).

c Particle size was measured using the Penn State Particle Size Separator (Nasco, Fort Atkinson, WI) as described by Kononoff et al. (2003).

**Table 6:** Final composition of diets fed during performance and apparent digestion trials in the second year.

**Item** ^a^	**TMR Performance** ^b^		TMR Digestion	
	NPR	PRO	NPR	PRO
**DM, % as fed**	35.7	36.9	35.6	35.7
**OM, % of DM**	91.5	91.6	90.2	90.3
**CP, % of DM**	11.8	11.8	12.7	13.4
**aNDF, % of DM**	41.5	41.2	45.4	42.6
**ADF, % of DM**	29.9	29.7	30.8	29.4
**Starch, % of DM**	26.9	27.9	25.9	24.4
**isSD 0 h, %**	13.3	30.4	–	–
**isSD 7 h, %**	35.7	42.2	–	–
**Lignin, % of DM**	5.7	6.7	5.7	5.5
**Fat, % of DM**	3.8	4.0	2.4	2.7
**Ash, % of DM**	8.5	8.4	9.8	9.7
**Ca, % of DM**	0.45	0.49	0.59	0.53
**P, % of DM**	0.19	0.19	0.20	0.22
**TDN, % of DM**	62.8	61.7	61.9	63.3
**NEm, Mcal/kg of DM** ^c^	1.33	1.30	1.30	1.34
**NEg, Mcal/kg of DM**	0.58	0.56	0.56	0.58

a Rock River Laboratory, Watertown-WI: DM, dry matter; OM, organic matter; CP, crude protein; EE, ether extract; aNDF, neutral detergent fiber using a-amylase; ADF, acid detergent fiber; isSD 0 h, ruminal in situ starch disappearance at 0 h, isSD 7 h, ruminal in situ starch disappearance at 7 h; Ca, calcium; P, phosphorus; TDN, total digestible nutrients.

b TMR, total mixed rations: NPR (non-processed) or PRO (processed) sorghum silage stored from 376 to 455d included at 90.5% diet; soybean expeller included at 6.5% diet; vitamin and mineral premix (ROC90, Provimi, Argentina), included at 3% diet; all on DM basis.

c NEm, net energy for maintenance; NEg, net energy for gain: estimated using equations from Galyean et al. (2016).

#### Laboratory analyses

Samples of feed ingredients as well as mixed diets were thawed and dried at 55°C for at least 48 h in a forced-air oven in the Universidad Nacional de la Plata, Buenos Aires, Argentina. Once dried, samples were re-weighed to calculate moisture content, and sent to Rock River Laboratory-Argentina (Santa Fe, Argentina) for quality determinations. All samples were ground in a Wiley mill (Arthur H. Thomas Co., Philadelphia, PA) through a 2-mm screen.

To determine sample OM concentration, approximately 0.5 g of the sample was weighed in duplicate, dried in a forced-air oven at 100°C for 24 h, and ashed at 550°C for 6 h following official method 990.03 (AOAC 1995). For determination of the fibrous component, samples were weighed in duplicate into F57 bags (Ankom Technology, Macedon, NY) and analyzed for neutral detergent fiber (NDF), using heat-stable α-amylase and sodium sulfite, and subsequently for acid detergent fiber (ADF) as described by Van Soest et al. (1991) in an Ankom 200 Fiber Analyzer (Ankom Technology). The concentration of CP was determined by Dumas combustion and nitrogen determination. (*N* × 6.25), in a Vario Micro Cube (Elementar, Manchester, U.K.) and a Leco TruMac N (Leco Corp., St. Joseph, MI); following official method 990.03 (AOAC 1995). Starch concentration in feed and feces was measured as proposed by Hall (2009). For in situ starch degradation, samples were sent to Rock River Laboratory (Watertown, WI) for analysis. Briefly, for in situ starch degradation, 3 g of sample dried and ground through a 6 mm sieve were weighed into R510 filter bags (5 × 10 cm, 50 ± 10 μm porosity; Ankom Technology) in triplicate. Bags were placed into weighted laundry bags and inserted into three cannulated lactating cows fed a conventional lactating diet for 7 hours. Digested bags were rinsed three times in cold water, dried overnight, and weighed back. Residues were ground to 1 mm and analyzed for starch concentration (Hall 2009). Fat concentration was determined by ether extraction following official method 920.39 (AOAC 2005). Finally, concentration of calcium and phosphorus in the diet was determined following official method 968.08 (AOAC 1995). Total digestible nutrients (TDN) was calculated using the OARDC (1998)/NRC (2001) summative approach described in NRC (2001). Tables 1 and 5 show the distribution of particles in the sorghum silage using the Penn State Particle Separator with sieves of 19.0, 8.0, 4.0 mm, and a bottom pan (Kononoff et al. 2003). The net energy for maintenance (NE_m_) and gain (NE_g_) for each diet was calculated using Lofgreen and Garrett (1968) equations from ME and performance Galyean et al. (2016):


$$\begin{array}{c} {\text{NE}}_{m}=\left(1.1104\times \text{ME}\right)\;–\;\left(0.0946\times {\;\text{ME}}^{2}\right)+\left(0.0065\times {\;\text{ME}}^{3}\right)\;–\;0.7783\\ {\text{NE}}_{g}=\left(1.1376\times \text{ME}\right)\;–\;\left(0.1198\times {\;\text{ME}}^{2}\right)+\left(0.0076\times {\;\text{ME}}^{3}\right)\;–\;1.2979\\ \text{Where}\;\text{metabolizable}\;\text{energy}\;\left(\text{ME}\right)=\left(0.9611\times \text{digestible}\;\text{energy}\right)\;–\;0.2999 \end{array}$$

#### Feeding trials

The first year feeding trial (80 d) consisted of 17 d adaptation period (d-17 to d-1) followed by 63 d growing period (d 0 to d 63). Shrunk liveweight (12 h feed restriction) was obtained on days −17, 0, and 63; additionally, interim unshrunk BW was obtained on d-30.

Second-year feeding trial (72 d) consisted of a 12-d adaptation period (d-12 to d-1) followed by a 60-d growing period (d 0 to 60). Shrunk liveweight was obtained on days −12, 0, and 60; additionally, interim unshrunk BW was obtained on d 33.

For each experiment, total BW gain was calculated as the difference between the final BW and the initial BW, and average daily gain (ADG) was calculated as total BW gain divided by the number of days on trial (63 and 60 d, for Y1 and Y2, respectively). Dry matter intake (DMI) was determined by the difference between feed offered and orts (that were not refed) with efficiency expressed as gain-to-feed ratio (GTF, kg/kg), computed as the ratio of ADG to daily DMI.

### Apparent total tract nutrient digestibility

When each performance study was finished, it was followed by an ATTD trial that was conducted from September 21 to 28, 2021 in Y1; and from August 8 to 15, 2022 in Y2. Because test diets for ATTD were the same as diets previously fed in the performance trials, each ATTD trial had only 3 d of adaptation (mainly to adapt heifer to being in the pen by themselves, without the rest of the heifers), followed by 4 d of an experimental period. Sorghum silage fed was the base diet fed in the performance trials, with the same storage time range as used in PE trials (<150 d in Y1 and > 375 d in Y2).

#### Experimental design and statistical analyses

From eight heifers/pen in twelve pens used for each performance trial, one heifer by pen was randomly selected to use in each digestion study. Data were analyzed as a CRD using the mixed model of SAS: fixed effect of treatment (SS either PRO or NPR), with initial BW used as a covariate. Significance was declared at *P* ≤ .05.

#### Diets and management in digestion trials

Randomly selected heifers were weighted to adjust the feed delivery of diets based on the average intake of the pen and the heifers’ weight, so that ad libitum intake was achieved as soon as possible with the single heifer per pen. Heifers selected to be enrolled in the digestibility trial remained in the same pen that they were housed during the growth performance study. Same ingredients (Tables 1 and 5) and composition (Tables 2 and 6), as used as in the performance studies, were fed ad libitum during 3 d of adaptation and 4 d of the experimental period (with feed and fecal samples collection). Day 0 was considered the beginning of the feeding of treatment diets (i.e., beginning of the adaptation). Feed was offered once daily during the ATTD collection period, and orts were collected the following day. To measure intake, feed offered, and orts were weighed using a portable scale (Mini Crane Scale OCS-L, IVS-engineering, Bulgary). Feed samples were collected from d 3 to d 7, and orts were collected from d 4 to d 8 to determine DM and nutrient composition. Fecal samples were collected twice daily (at 0800 and 1900 h) for 4 consecutive days (d 4 to d 8) directly from the intact feces found on the pen surface, avoiding contamination with dirt from the pen.

#### Digestibility and laboratory analyses

The apparent total tract digestibility of nutrients was determined using iNDF as an internal marker. Immediately after being collected, feed, orts, and fecal samples from each heifer at each time point were individually stored and frozen at −20°C; feed and orts samples were weighed before being stored in the freezer. At the end of the experiment, all samples were thawed and dried at 55°C for at least 48 h in a forced-air oven in the Universidad Nacional de la Plata, Buenos Aires, Argentina. Once dried, feed and ort samples were re-weighed to determine the moisture content, and sent to Rock River Laboratory (Santa Fe, Argentina) for further analyses. All samples were ground in a Wiley mill (Arthur H. Thomas Co., Philadelphia, PA) through a 2-mm screen. Individual feed, orts, and fecal samples were composited per heifer on an equal dry matter weight basis to determine nutrient and marker concentrations.

To analyze concentrations of iNDF in feed and feces, 1 mm ground samples were digested in vitro with a standardized rumen inoculum at 39°C under continuous carbon dioxide for 240 h (Rock River Laboratory). Concentrations of OM, NDF, ADF, CP and starch in feed and feces were determined as previously described. Apparent total tract digestibility of DM, OM, NDF, ADF, CP and starch were calculated using the following formula:


$$100-100\;\times [\left(\frac{\mathrm{iNDF}\;\mathrm{concentration}\;in\;\mathrm{feed}}{\mathrm{iNDF}\;\mathrm{concentration}\;in\;\mathrm{feces}})\times (\frac{\mathrm{nutrient}\;\mathrm{concentration}\;in\;\mathrm{feces}}{\mathrm{nutrient}\;\mathrm{concentration}\;in\;\mathrm{feed}}\right)]$$

The percentage difference of NDF concentration between diet and refusals (change on NDF) was calculated using the following formula:


$$100\times (\frac{\mathrm{NDF concentration}\;in\;\mathrm{refusals}-\mathrm{NDF concentration}\;in\;\mathrm{diet}}{\mathrm{NDF concentration}\;in\;\mathrm{diet}})$$

### In situ degradation trial

From sorghum harvested on May 17, 2021, composite samples from each SS treatment (NPR and PRO) were taken on days 0, 66, 157, 202, 246, 291, 381 and 455, and frozen until September 2022. Samples were thawed and dried at 55°C for at least 48 h in a forced-air oven in the Universidad Nacional de la Plata, Buenos Aires, Argentina. Once dried, samples were not ground to avoid affecting treatments, and sent to North Florida Research and Education Center, Marianna, FL, 32446, USA; for the in-situ trial.

#### Ruminal degradation

Ruminal in situ degradation of silage treatments by sampling day, was determined in duplicate using four ruminally cannulated steers [423 ± 35 kg body weight (BW); average BW ± SD] that had consumed corn silage base diets for at least 15 d. Dried, unground samples of NPR and PRO SS, identified by sampling day were weighed (5 g) into 10 × 20 cm Ankom in situ bags (R1020, Ankom Technology Corp., Macedon, NY). The bag pore size was 50 μm and the sample size to free bag surface area ratio was 12.5 mg/cm^2^. Bags were heat-sealed and placed in zippered mesh bags attached to a rope and carabiner, weighed, immersed in warm (39°C) water for 15 min, and incubated in the ventral sac of the rumen. All bags were placed at the same time in the rumen and removed after 24 h. After removal, all bags were placed in a cooler with ice water to halt fermentation, subsequently rinsed with cold running tap water to remove adherent particles and bacteria, frozen overnight, and then manually rinsed without soap until obtaining clear water. The same rinsing procedure was applied for bags not incubated in the rumen. Rinsed bags were dried for 48 h at 55°C and weighed. After weighing, residues from the ruminal incubations (2 bags/time point/steer/treatment) were composited by incubation time within steer. Composited samples and original whole samples were analyzed for determination of DM and starch to calculate 24-h ruminal degradation of each component. Laboratory Analyses were made by Rock River Laboratoy.

#### Statistical analysis

Data were analyzed as a generalized randomized block design with repeated measures. The fixed effect of treatment, day and their interaction were included in the model; the repeated subject was considered the animal within treatment. Significance was declared at *P* ≤ .05.

## Results and discussion

Sorghum silage yield from the 11.2 hectares used for the study averaged 37.25 tons of greenchop/ha, and the packing density achieved in the ag-bags was 3600 kg/linear m (average for both processing treatments). The average yield achieved with sorghum silage in this study highlights the capacity of this crop to produce sufficient forage despite limited rainfall (only 380 mm during the growing season for this study), an amount considered insufficient to ensure an adequate corn yield. As observed in Table 1, processing sorghum at harvest (SSPRO) led to a greater proportion of fine particles as shown by the Penn State Particle Separator (46.1% ≤ 1.18 mm), when compared with non-processed sorghum silage (SSNPR; 38.8% ≤ 1.18 mm). A similar impact of processing in reducing particle size and increasing silage packing density was reported by Johnson et al. (2002), working with corn silage.

Composition of the total mixed rations fed, based on laboratory analysis (both in performance and ATTD trials on Y1), are presented in Table 2. In situ starch disappearance in the diet was greater for PRO by 30.1 and 9.1 percentage units at 0 and 7 h of ruminal incubation, respectively, suggesting a positive effect of processing on exposing more starch for digestion in SS. While this is a characterization of the diet fed and not statistically analyzed, the fact that despite the grinding of samples for this assay there is such a difference in starch disappearance is noteworthy.

Performance results of Y1 are presented in Table 3. There were no effects of treatment on DMI, either expressed in kg/d, or as % of BW (*P* ≥ .24). Initial BW was not different among treatments (*P* = .99), whereas the final BW and ADG were greater (*P* < .02) for the PRO treatment. Finally, heifers in PRO treatment were more efficient in feed conversion (0.128 vs. 0.107 GTF for PRO and NPR, respectively; *P* = .03). Processing sorghum silage at harvest increased ADG by 15%. Even though no differences were detected for DMI, the additional (numerical) 0.25% of BW consumed by heifers in the NPR treatment, together with their lesser ADG led to the 21% decrease in feed efficiency in the NPR treatment. It is noteworthy that these results were achieved using SS with less than 150 d of storage (47 to 134 d of storage during Y1), which represents the most typical silage storage length by beef producers using silage as a winter-feeding strategy.

Table 2 shows dietary NEm and NEg concentrations calculated from laboratory analyses in Y1, using calculated TDN using the summative approach described in NRC (2001) and actual DMI, (Table 3), with shrunk ADG calculated using equations from Lofgreen and Garrett (1968), adjusted by Galyean et al. (2016). Calculated ADG based on the described equations were 0.96 and 0.99 kg for NPR and PRO, respectively (data not shown), and those differed slightly from actual ADG (Table 3). Differences between calculated and observed ADG may be attributed to the impact of processing SS. Equations based on laboratory analyses of ground samples may not fully account for the effect of processing on starch digestibility in the whole tract. Data from the ATTD trial in Y1 (Table 4) provides strong supporting evidence to the effect of kernel processing on nutrient digestibility and its impact on animal performance. During the ATTD trial, intake of OM tended (*P* < .07) to be greater for NPR, while intake of NDF, ADF and CP did not differ between treatments (*P* ≥ .10). Starch intake for heifers in Y1 increased (*P* = .03) by 2.54 kg/d in NPR (Table 4). The lack of differences in intake of NDF and ADF intake between both treatments implies that ruminal and digestive tract fill was the primary factor limiting intake of DM in these diets. An increase (*P* < .01) of 95% in fecal starch concentration was observed for NPR (8.46%) when compared to PRO (4.46%). This resulted in a 7.6% decrease (*P* < .01) in apparent total digestibility of starch in NPR when compared with PRO (86.48% and 93.55%, respectively; Table 4).

Although starch intake was greater for cattle fed the NPR treatment (Table 4), this was not sufficient to enhance ADG performance, likely because of the dietary starch of the NPR treatment was less digested when sorghum silage was not processed. Same results were reported by Podversich et al. (2025), evaluating processing type on whole plant sorghum silage. Conversely, kernel processing had a positive impact on ATTD of starch in PRO, increasing by 8%, which partially explains the greater ADG in this treatment (106 g/d more than in NPR) during Y1. This additional ADG could not be predicted from the dietary NEg concentrations calculated from laboratory assays (Tables 1 and 2) and using the Lofgreen and Garrett (1968) equations, with corrections by Galyean et al. (2016). This has to be considered when formulating growing diets using sorghum silage and using laboratory data (generated from ground samples) to estimate growth performance.

Tables 5–8 show data from the Y2 trials, when silage fed was stored for more than 375 d. When comparing the analyzed nutritional composition of diets fed in Y1 (Table 2) and Y2 (Table 6), it can be observed that DM content of the diet, starch and NDF concentrations were quite similar across years. Even calculated values of energy density (i.e., NEm and NEg) remained fairly constant across years. However, the same consideration as discussed previously should be applied to the accuracy of laboratory-based calculations of energetic density in sorghum silages.

**Table 7:** Effects of processing sorghum silage at harvest, on performance of beef heifers during the second-year trial.

**Item** ^a^	**Treatments** ^b^		**SEM** ^c^	***P-*value** ^d^
	NPR	PRO		
**Pens, n**	6	6	**-**	**-**
**DMI, kg/d**	8.08	8.81	0.168	0.01
**DMI, % of BW**	3.42	3.69	0.08	0.03
**Initial BW, kg**	198.7	197.4	10.01	0.93
**Final BW, kg**	248.6	250.6	1.80	0.46
**ADG, kg**	0.830	0.902	0.04	0.22
**GTF, kg: kg**	0.103	0.103	0.004	0.96

a DMI, dry matter intake; BW, body weight; ADG, average daily gain; GTF, gain to feed ratio on a DM basis.

b Treatments: sorghum silage stored from 376 to 455 d non-processed (NPR) or processed (PRO) included at 90.5% of total diet; soybean expeller at 6.5% of total diet; Premix (ROC90, Provimi, Argentina) at 3% of total diet; all on DM basis.

c Standard error of the mean (*n* = 6 pens/treatment).

d Observed significance level for treatment (*n* = 6 pens/treatment).

**Table 8:** Effects of processing sorghum silage on intake and total tract digestibility of backgrounding beef heifers during the second-year trial.

**Item** ^a^	Treatments^b^		**SEM** ^c^	***P-*value** ^d^
	NPR	PRO		
**Heifers, n**	6	6	*-*	*-*
**Body weight, kg**	250.6	248.6	1.803	0.46
**Intake, kg/d**				
**DM**	7.78	8.11	0.491	0.65
**OM**	7.03	7.32	0.446	0.65
**NDF**	3.54	3.44	0.216	0.76
**ADF**	2.39	2.38	0.165	0.94
**CP**	0.99	1.08	0.068	0.37
**Starch**	2.01	1.99	0.160	0.94
**Change in NDF, %^e^**	6.04	12.37	2.240	0.07
**Fecal starch, %**	7.28	7.55	0.688	0.79
**Digestibility,%**				
**DM**	48.72	49.50	4.119	0.89
**OM**	51.64	52.86	3.900	0.83
**NDF**	32.76	35.69	3.066	0.54
**ADF**	16.75	21.63	4.551	0.49
**CP**	43.30	48.81	4.521	0.41
**Starch**	85.49	83.91	2.368	0.65

a DMI, dry matter; OM, organic matter; NDF, neutral detergent fiber; ADF, acid detergent fiber; CP, crude protein.

b Treatments: sorghum silage stored from 376 to 455 d non-processed (NPR) or processed (PRO) included at 90.5% of total diet; soybean expeller at 6.5% of total diet; Premix (ROC90, Provimi, Argentina) at 3% of total diet; all on DM basis.

c Standard error of the mean (*n* = 6 pens/treatment).

d Observed significance level for treatment (*n* = 6 pens/treatment).

e Change in NDF, % = (NDF concentration refusals—NDF concentration diet)/NDF concentration diet × 100.

Animal performance results for Y2 are presented in Table 7. No effects of treatment (*P* ≥ .22) were observed on ADG, initial or final BW, or GTF, even though processing sorghum silage at harvest increased DMI, both in kg/d (*P* = .01) or as % of BW (*P* = .03). While statistically significant, the extra 73 g/d of DMI in PRO was not sufficient to significantly impact ADG and instead resulted in a similar FE when compared to the NPR treatment (*P* = .96) in Y2. While a direct comparison between Y1 and Y2 performance studies is not possible due to the confounding effect of year, previous data shows that a longer ensiling length in sorghum silage enhances starch disappearance, at least when measured in situ and with up to 90 d of storage (Fernandes et al. 2020; Duhatschek et al. 2025). The Lofgreen and Garrett (1968) equations, corrected by Galyean et al. (2016) were applied in Y2 to estimate ADG based on tabular (laboratory analyses) energy density values for each diet, and using ingredient analyses (Tables 5 and 6), animal weight and feed intake recorded (Table 7). Calculated ADG was slightly greater (0.94 and 0.99 kg for NPR and PRO diets, respectively; data not shown) than the observed live performance results, maintaining numerical differences in favor of the PRO treatment (+ 50 g/d). Despite this, even the numerical 70 g of observed ADG in favor of PRO were not sufficient to detect a difference between treatments (*P* = .22; Table 7).

The lack of differences due to processing in Y2 could be explained by the ATTD trial results presented in Table 8. There was no effect (*P* ≥ .37) of treatment on intake of DM, OM, NDF, ADF, CP and starch. The lack of differences in daily ADF intake for NPR and PRO diets suggests again that gut fill and capacity to remove ADF from the rumen via digestion perhaps limited dry matter intake in both diets. Likewise, extent of apparent total tract digestion of DM, OM, NDF, ADF, CP and starch were not altered (*P* ≥ .41) by kernel processing in Y2 when the SS was stored for over 375 d (Table 8). Lack of differences in nutrient intake and digestion are in perfect agreement with the lack of effects on growth performance variables found among treatments in Y2. While changes in feed intake were not evident during the ATTD trial in Y2, heifers fed the PRO diet showed a tendency (*P* = .07) to sort out and refuse to eat a greater proportion of the dietary NDF (Table 8). With less fiber in the consumed diet, a lesser ruminal fill and greater passage rate could be expected, as reported by Krizsan et al. (2010), helping perhaps to understand the greater feed intake recorded for PRO during the Y2 performance trial (Table 7).

Most of the improvements on starch digestion associated with silage storage were apparent when the grain was processed prior to ensiling, with the main benefits occurring during the first 30 days of storage. Increases in starch digestibility of high-moisture corn continue over time, as shown in the meta-analysis by Daniel et al. (2015). Presumably this increase is due to fermentation acids solubilizing prolamines that are hydrophobic and inhibit microbial attachment and digestion of starch.

Reports by Saylor et al. (2021), showed that with short storage, there were limited benefits in starch digestion when the grains remained intact in the silage. It is interesting to highlight that even with SS, where whole grains are difficult to digest; a longer storage time as reported in this study (>375 d), still may show benefits in terms of enhanced starch degradability. Differences on animal performance and digestion due to processing SS with short storage time (≤150 d), were not detected with long storage when SS was fed as the base of backgrounding diets. Diepersloot et al. (2022) and Saylor et al. (2021) have demonstrated that storage time increases starch digestibility in corn silage, and this seems to be mediated by the effects of protelytic bacteria, including those that may be applied during silage inoculation (Junges et al. 2017). Moreover, recent work by (Duhatschek et al. 2025) shows that increasing whole-plant sorghum ensiling time from 0 to 90 days, increased starch digestibility when harvested using a conventional kernel processor, but not when harvested without a kernel processor. This concept is also supported by Fernandes et al. (2020) when measuring in situ starch disappearance in sorghum silage stored for up to 90 d. It should be noted that there are no studies in the literature testing sorghum silage when ensiled for more than a year, with or without kernel processing at harvest. Thus, the findings by Duhatschek et al. (2025) are in agreement with our Y1 data, showing conclusive proof of the effects of kernel processing but only up to 90 d of storage.

**Figure 1:**
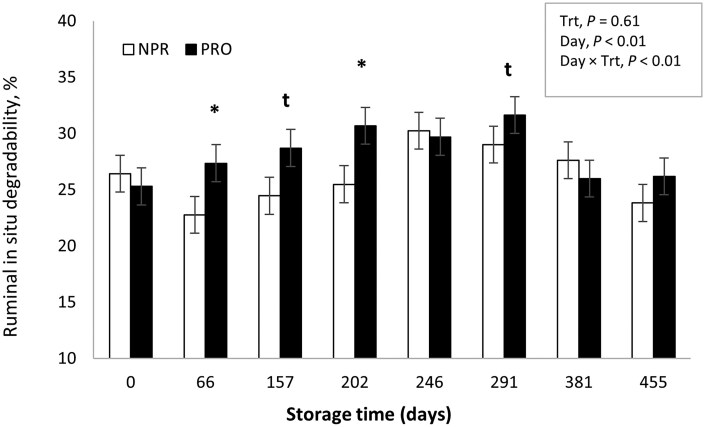
Effect of processing and storage time on ruminal in situ dry matter degradability of sorghum silage incubated for 24 h. Trt = treatment; NPR = non-processed sorghum silage, PRO = processed sorghum silage. Significance level for treatments within day: * = *P* < .05; t = 0.07 *≤* *P* ≤ .11.

**Figure 2:**
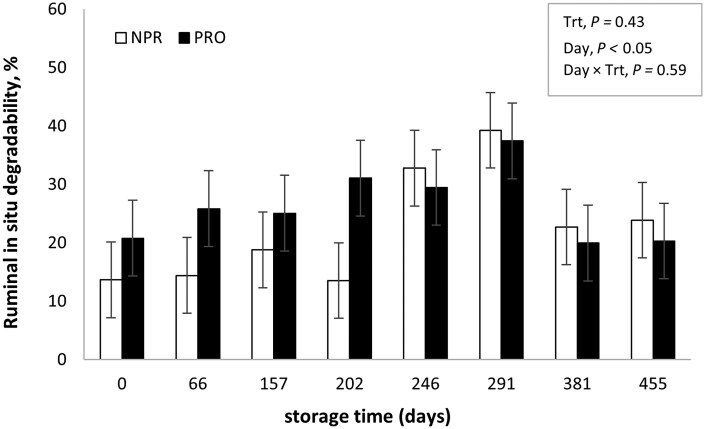
Effect of processing and storage time on ruminal in situ starch degradability of sorghum silage incubated for 24 h. Trt = treatment; NPR = non-processed sorghum silage, PRO = processed sorghum silage.

**Figure 3:**
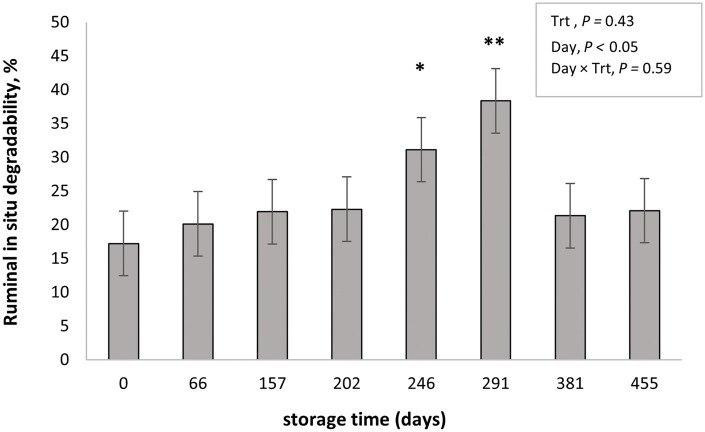
Effect of storage time on ruminal in situ starch degradability of sorghum silage processed or not at ensiling time and incubated for 24 h. Trt = treatment. Significance level of day when compared to day 0: * = *P* < .05, ** = *P* < .01.

The in situ experiment was conducted with samples taken in parallel to the performance and ATTD trials carried out across the two years, and the purpose was to generate additional information to help understand and integrate results of these experiments. However, the authors are aware of its limitations in terms of potential sampling and laboratory errors. Nevertheless, the findings from our in situ study, taken together with previous studies conducted with this methodology (Fernandes et al. 2020; Duhatschek et al. 2025) shed light on the potential mechanisms behind the animal performance responses. Figure 1 shows the effect of treatment (PRO or NPR) and storage time on in situ rumen DM disappearance of SS at 24 h of incubation. A day × treatment interaction was detected for in situ DM degradability (*P* < .001). The PRO treatment increased (*P* < .05) in situ DM disappearance at d 66 and 202 of storage and tended (*P* > .07) to increase it at d 157 and 291; showing a positive effect of processing up to ≤ 300 d of storage. This interaction between storage time and processing implies that beyond 291 d of storage, the effect of kernel processing is greatly reduced, as evidenced by both the in situ DM disappearance and the Y2 animal performance data. These results are in agreement with previous studies using corn and sorghum silage, where positive effects of short-term storage occurred with processed grains (Fernandes et al. 2020; Saylor et al. 2021). It is likely that this effect of storage time on DM degradability observed by many authors, was partially responsible for the results during our Y1 studies, where processing improved performance and digestion when sorghum silage was fed for up to 134 d of storage. In situ starch disappearance is shown in Fig. 2. Although starch degradation curves followed a similar pattern to DM, perhaps due to a greater variability in starch concentration across sampling period, no effect of treatment (*P* = .43) or treatment × storage time interaction (*P* = .59) were detected. An effect of storage time on starch degradability was detected (*P* < .05), thus, in Fig. 3 treatment means are presented across storage days to show the time effect. No differences in storage time on in situ starch disappearance were detected until d 202 when compared to d 0 (*P* ≥ .10; Fig. 3). Between d 202 and 291, starch disappearance increased (*P* < .05), with no differences detected from d 381 to the end of storage (d 455) when compared with d 0.

The positive effects of storage time on in situ disappearance of DM and starch, always compared to d 0, were lost over 300 d in our study, regardless of processing, returning to initial values of in situ degradability reported from d 0 to 202. It is difficult to explain those findings, since most in vitro studies show that improvements in starch degradability continues over time but at a lesser rate, as shown in the meta-analysis by Daniel et al. (2015). It could be speculated that the type of material used for conservation (e.g., permeability of the ag bag, etc.) could have affected the ensiled forage over time. Ag bags used in this experiment were the thinnest available in the market (220 µm), in contrast with plastics of greater than 300 µm that can improve oxygen impermeability. Nevertheless, we did not detect any problems related with molding or SS acceptability by the heifers.

Overall, processing sorghum silage at harvest had positive impact on improving performance and apparent starch digestion in the total tract, when the SS was stored for a < 150 d. When a sorghum silage-based diet was fed to growing beef heifers after more than one year of storage, kernel processing does not seem to affect either cattle performance or digestibility of nutrients in the total tract. Although the two performance experiments were conducted with different animals and in two consecutive years, the same cattle genetics, time of year, facilities, and harvested silage (comprising 90.5% of the diet DM in both years) were used. Yet, it is impossible to disentangle the effects of silage storage length from the effect of year, and caution needs to be exercised when comparing the two studies. The overall mean ADG obtained in Y2 was 10% less than in Y1 while, again, not directly comparable. The lower in situ degradability of DM and starch observed after 300 d of storage, plus the reduced DM and starch digestibility observed in Y2 (Table 8) vs. Y1 (Table 4) could be partially responsible for the differences in ADG. This observation is in agreement with the suggestion of Temitope et al. (2022), who showed that prolonged storage of silages increased nutrient losses and hence reduced expected performance in ruminants when silage was the main ingredient in the diet. From a practical standpoint at the farm level, and from an economical perspective, it seems difficult to justify storing silages longer than one year prior to feeding. However, it is known that some dairies in the U.S. do store silages for that long, in an attempt to increase starch digestion and available energy content of silages as long as feed inventory allows it.

## Conclusions

Heifers fed sorghum silage processed at harvest and stored for less than 150 d, gained weight faster, converted feed to gain more efficiently and digested starch more completely than heifers fed non-processed sorghum silage. Conversely, when sorghum silage was stored for more than one year (>375 d to be precise), no differences in average daily gain, feed conversion and nutrient digestion were detected among treatments. Processing sorghum silage at harvest, increased ruminal in situ DM degradability until 300 d of storage. Starch degradability increased with storage time regardless of kernel processing. Lack of differences over 300 d of storage in DM and starch degradation in situ are in agreement with the lack of differences in animal performance and apparent total tract nutrient digestibility observed when sorghum silage was stored over 375 d and fed to backgrounding heifers. Future work should focus on the mechanisms by which starch digestion is impacted with increased time of storage. In particular, research is needed on the role of prolamines in delaying starch degradation and potential factors that affect their solubility during the ensiling process.

## Abbreviations

ADF: acid detergent fiber
ADG: average daily gain
ATTD: apparent total tract digestibility
BW: body weight
Ca: calcium
CP: crude protein
DM: dry matter
DMI: dry matter intake
EE: ether extract
GTF: gain to feed ratio
ISND: in situ nutrient degradability
NEg: net energy for gain
NEm: net energy for maintenance
aNDF: neutral detergent fiber using α-amylase
NPR: not-processed
OM: organic matter
PRO: processed
P: phosphorus
SS: sorghum silage
TDN: total digestible nutrients
iNDF: indigestible NDF
Y1: first year
Y2: second year
